# Fear and loathing in the Caribbean: three studies of fear and cancer screening in Brooklyn's immigrant Caribbean subpopulations

**DOI:** 10.1186/1750-9378-4-S1-S14

**Published:** 2009-02-10

**Authors:** Nathan S Consedine, Brenda A Adjei, David Horton, Andrew K Joe, Luisa N Borrell, Paul Michael Ramirez, Tracey Ungar, James M McKiernan, Judith S Jacobson, Carol Magai, Alfred I Neugut

**Affiliations:** 1 Department of Psychology, Long Island University, Brooklyn, NY 11201, USA; 2 Intercultural Institute on Human Development & Aging, Brooklyn, NY 11201, USA; 3 Herbert Irving Comprehensive Cancer Center, Columbia University Medical Center, New York, NY 10032, USA

## Background

Anxiety, worry, and fear are among the most common emotional responses to the threat of disease and several studies have linked various fears to cancer preventive and detection behaviors. Cancer-related worry and fears about screening or its consequences are also characteristics that vary across ethnic groups and may be differentially linked to screening outcomes [1]. Limiting the utility of this growing literature are at least two key considerations.

First, little attention has been paid to documenting variation in cancer-related fears among subpopulations of persons of African descent, despite evidence that (a) rates of screening may vary among both male [2] and female [3] immigrants from islands in the Caribbean living in the United States and (b) incidence rates for cancers such as those of the prostate may be very high in men from Jamaica [4], Guadaloupe [5] and Trinidad and Tobago [6], as well as in immigrant groups in both the United Kingdom [7] and United States [8]. Second, findings regarding the relations anxiety, cancer worry, and screening fear hold with screening behavior seen thus far have been inconsistent, in our view because anxieties stemming from different sources have different relations with behavior. In the emotions theory view, understanding the role of fear in health behavior in diverse groups is predicated on understanding the *object *or *source *of the fear [9-11] for the simple reason that anxiety motivates avoidance of particular elicitors [10,12].

Research conducted within the U54 Comprehensive Cancer Partnership between Long Island University and Columbia University has produced several studies documenting differences in breast and prostate cancer screening frequencies among Caribbean subpopulations living in Brooklyn, New York [1,12,13]. A major concentration in this program of behavioral research has investigated whether trait anxiety, cancer worry, and screening-related fears vary across Caribbean subpopulations and whether these highly differentiated emotional responses independently predict screening behavior in multivariate models [1,2,12-14]. Consistent with theory, we expected that fears pertaining to the screening context (e.g., fear of pain or the psychological implications of certain screens), would predict avoidance of the fear-inducing situation and thus be associated with less frequent screening. Conversely, where fears relate to the disease itself, greater fear should predict more frequent screening.

## Methods

Because of our overarching interest in the links between cancer and cancer-screening-related fears and cancer screening behaviors among the diverse groups of men and women living in Brooklyn, New York, we combined data from three community-based studies. Although measures and samples varied somewhat across studies, each study investigated the link between emotions and screening outcome in ethnic groups that included immigrants from islands in the Caribbean. Because of our interest in examining differences *within *traditional racial categories, we used a combination of (a) self-categorization based on a the traditional racial categories offered in the US Census together with (b) information regarding country of origin. Allowing a combination of self-reported racial categorization (tapping aspects of identity and minority status) in concert with shared birthplace to influence groupings increases the likelihood that participants share cultural and developmental characteristics thought to form part of ethnicity [15]. We distinguished between Black men born in the United States (hereafter, U.S.-born African Americans), and those originating from countries in the English-speaking Caribbean (e.g., Trinidad & Tobago, Jamaica, Barbados). Immigrant and non-immigrant minority groups were contrasted with men self-identifying as "European or White/Non-Hispanic" who were born in the United States (hereafter, U.S.-born European American).

In Study 1, stratified cluster-sampling was used to recruit 1364 women (aged between 50–70 years) from six ethnic groups: US-born African American, US-born European American, immigrants from islands in the English-speaking Caribbean (Jamaica, Barbados, Trinidad and Tobago), the Dominican Republic, Haiti, and Eastern Europe [1]. In Study 2, 180 US-born African American, US-born European American and immigrant Jamaican men (aged between 40–70 years) were recruited using convenience sampling [13]. In Study 3, 533 men (aged between 45–70 years) from four groups – US-born African American, US-born European American, and immigrant men from Jamaica and from Trinidad and Tobago – were recruited [12]. In each study, participants provided background data, reported on screening history for either breast or prostate cancer, and completed a measure of trait anxiety, cancer worry, and/or screening fears.

## Results

**Figure 1:**
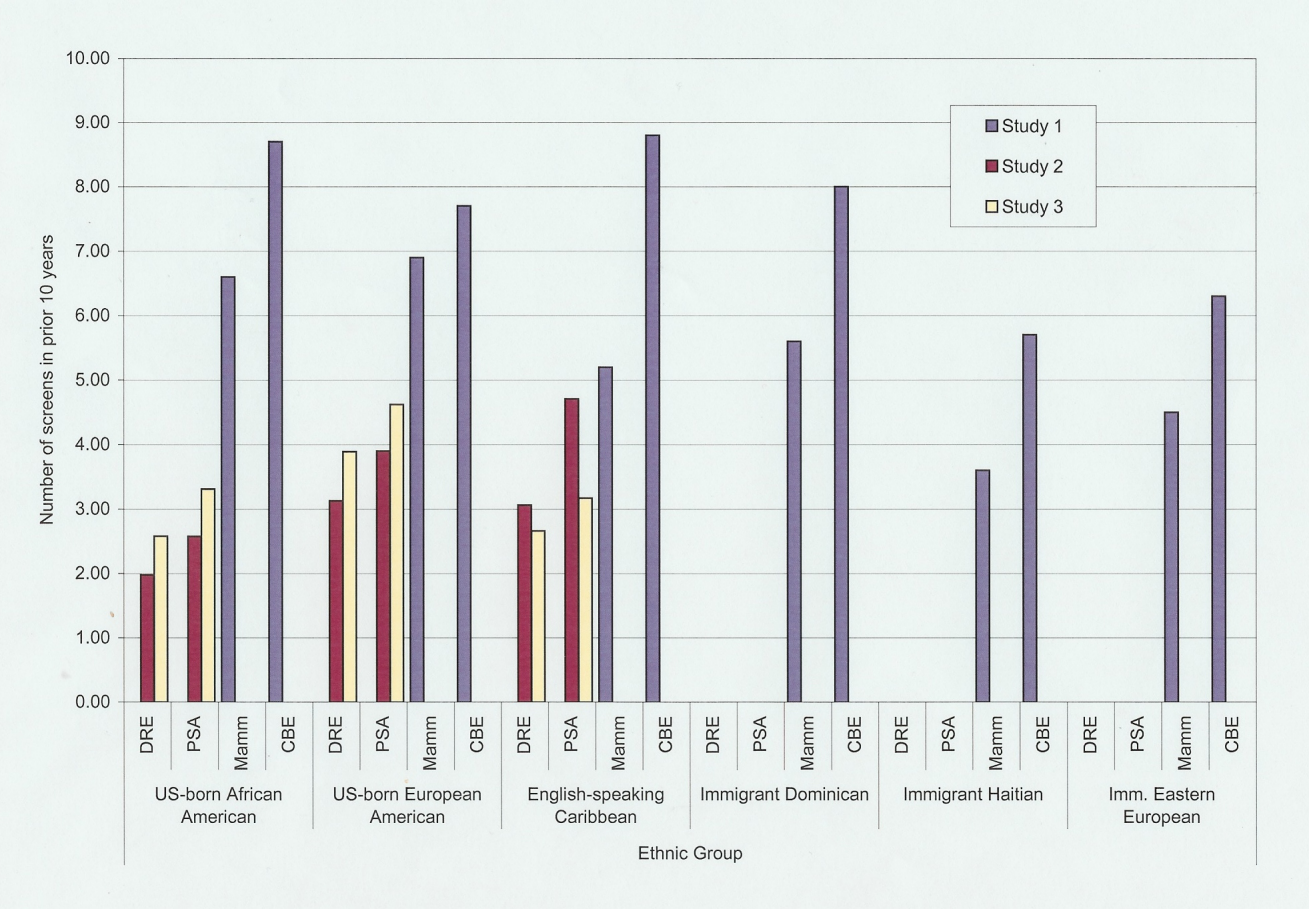
**Number of cancer screens in prior 10 years**. DRE = digital rectal examination, PSA = prostate specific antigen test, Mamm = mammogram, CBE = clinical breast exam.

**Figure 2:**
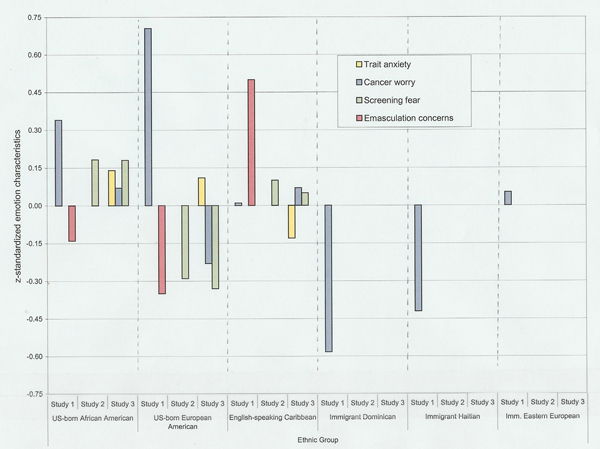
**Emotion characteristics related to screening**. Trait = trait anxiety, Worry = cancer worry, Scr. Fr. = screening fear, and Em. Con. = emasculation concern.

As expected, we found differences among groups of African descent from the United States and the Caribbean. Although women from all groups screened at rates below those recommended, data from Study 1 showed that English-speaking Caribbean, Haitian and Dominican women screened less frequently than US-born African Americans and European Americans and that immigrant Eastern European women were also infrequent screeners (see Figure 1). Conversely, however, there were no differences in rates of self-reported *prostate *screening among men from the English-speaking Caribbean, US-born African Americans, or US-born European Americans in either Study 2 or Study 3. As expected, cancer-related emotional characteristics also varied across subpopulations (see Figure 2). Cancer worry was generally lower among women from the various Caribbean immigrant groups (Study 1) than it was among US-born African Americans or US-born European Americans. Fears regarding screening, however, varied somewhat differently. Fear of screening was higher among US-born African Americans and immigrant men from the English-speaking Caribbean (Studies 2 and 3) than among US-born European Americans. Consistent with the need to carefully measure fear-related constructs in the context of cancer behavior, however, our data also demonstrated that a specific fear related to concerns regarding threats to masculinity in the context of male screening strongly characterized the attitudes of men from the English-speaking Caribbean compared to the views of US-born European and US-born African Americans (Study 2). Finally, a combination of multiple regression and ANOVAs in each study showed that emotional characteristics independently predicted screening, in most cases even when background characteristics were controlled. Across studies, greater cancer worry predicted more frequent screening while fear of screening predicted less frequent screening.

## Conclusion

Data from three large-scale studies in Brooklyn, New York suggest that members of immigrant Caribbean subpopulations screen for breast and prostate cancer at very low rates; in most cases lower than those of either US-born African or US-born European Americans. Groups of Caribbean men and women also vary in the emotions they report regarding cancer and the screening process, generally revealing a pattern that is predictive of poorer screening. Coupled with the fact that emotion characteristics predicted screening outcomes even when controlling for other factors, data from these three studies suggest that the emotional responses Caribbean groups place them at risk for poor screening. Interventions that address these responses may offer the prospect of improving screening frequency in these disadvantaged groups.

## Competing interests

The authors declare that they have no competing interests.

## Authors' contributions

NSC and CM was involved in study design, analysis, interpretation/write up and critical revision of the manuscript.

BA and DH in the analysis, interpretation and write up. AKJ, TU and LNB were involved in the interpretation and write up. PMR was part of the study design, analysis and interpretation/write up. JMM and AIN had part in the study design, analysis and critical revision whilst JSJ took part in critical revision.
